# Paraneoplastic secondary hypertension due to a renin-secreting desmoplastic small round cell tumor: A case report

**DOI:** 10.3892/ol.2014.2452

**Published:** 2014-08-18

**Authors:** HEE-JEONG LEE, JIN-SOO HYUN, HOE-SOO JANG, HYOUNG SUL, SANG-GON PARK

**Affiliations:** Department of Internal Medicine, Chosun University Hospital, Gwangju 501-717, Republic of Korea

**Keywords:** desmoplastic small round cell tumor, aldosterone, plasma renin activity, paraneoplastic secondary hypertension, renin-producing tumor

## Abstract

Desmoplastic small round cell tumor (DSRCT) is a rare and aggressive malignancy with a poor outcome that occurs in adolescents and young adults; <200 cases of DSRCT have been reported. Renin-producing tumors are also rare and cases of extrarenal renin-producing tumors are even rarer. The present study describes the case of a 20-year-old male that was diagnosed with DSRCT and presented with severe hypertension and hypokalemia, as well as metabolic alkalosis. The plasma renin activity (PRA) level was identified to be markedly elevated (normal range in standing and supine positions, 1.3–4.0 ng/ml/h and 0.15–2.33 ng/ml/h, respectively) and the plasma aldosterone level was also increased (normal range in standing and supine positions, 4.0–31.0 ng/dl and 1.0–1.6 ng/dl, respectively). The symptoms of the patient were consistent with the renin-secreting tumor triad, which comprises hypertension, hypokalemia and elevated PRA. Paraneoplastic syndromes must always be considered in cancer patients exhibiting unusual clinical findings, despite their rarity. The current patient was diagnosed with paraneoplastic secondary hypertension due to the presence of disseminated renin-secreting DSRCT. The patient was treated with the VAC/IE regimen (vincristine, adriamycin, cyclophosphamide, ifosfamide and etoposide) for six cycles. Following this treatment, the serum renin and aldosterone levels fell to within the normal range and the patient’s blood pressure was normalized without antihypertensive medication. Although an immunohistochemical evaluation of renin was not conducted as the sample size was inadequate, the present study demonstrated that the tumor had produced renin. The biosynthesis of renin was identified by the presence of mRNA that coded for the renin precursor, which was observed in the ascites of the patient. The current study describes, to the best of our knowledge, the first reported case of paraneoplastic secondary hypertension in a patient presenting with a renin-producing DSRCT.

## Introduction

Desmoplastic small round cell tumor (DSRCT) is a rare type of mesenchymal tumor that was first described as a separate tumor type in 1989 by Gerald and Rosai (1). Worldwide, <200 cases are reported in the literature. DSRCT typically affects adolescents and young adults (predominantly males) and develops in the abdominal cavity. Despite the availability of multimodal treatment, DSRCT has a highly aggressive clinical course. In addition, the majority of patients experience resistant and recurrent disease as they approach the end of life, the three-year survival rate is 44% and the five-year survival rate is 15% (2). However, >50% of the patients reported by Lal *et al* (2) did not exhibit distant metastasis. There is no standard therapy for patients with DSRCT, particularly for those patients with metastatic DSRCT, and there are few reports of metastatic DSRCT treatment. Kushner *et al* (3) reported 12 DSRCT patients, with a median survival time of 19 months. A patient reported by Mrabti *et al* (4) has a survival period of 26 months following the diagnosis of DSRCT.

Renin-producing tumors are rare, and cases of extrarenal renin-producing tumors are particularly rare. The current study presents a case of a renin-producing DSRCT. Written informed consent was obtained from the patient’s family.

## Case report

In January 2011, a 20-year-old male was admitted to the Department of Internal Medicine, Chosun University Hospital (Gwangju, Korea) with a complaint of abdominal distension and a palpable mass in the abdomen. The symptoms had begun two months previously and the palpable mass had gradually grown over the two weeks prior to presentation. The patient’s personal and family medical histories were non-specific with the exception of high blood pressure (BP; 150/100 mmHg; normal range, 100–120/70–80 mmHg).

The patient’s vital signs were as follows: Body temperature, 36.6°C (normal range, 36.5–37.5°C); BP, 180/110 mmHg; pulse, 108 beats per min (normal range, 60–100 beats per min); and respiratory rate, 18 breaths per min (normal range, 12–20 breaths per min).

Physical examination revealed a 1-cm non-tender, hard and fixed nodule surrounding the umbilicus with abdominal distension and without fluid shifting.

The laboratory results were as follows: White blood cell count, 5,740/mm^3^ (normal range, 4,000–8,000/mm^3^); hemoglobin, 14.3 g/dl (normal range, 14.0–18.0 g/dl); platelet count, 282×10^3^/mm^3^ (normal range, 150–400×10^3^/mm^3^); total protein, 7.83 g/dl (normal range, 5.3–7.4 g/dl); albumin, 4.49 g/dl (normal range, 3.5–5.2 g/dl); aspartate aminotransferase, 19 IU/l (normal range, 5–40 IU/l); alkaline aminotransferase, 14 IU/l (normal range, 5–40 IU/l); alkaline phosphatase, 115 IU/l (normal range, 35–123 IU/l); serum lactate dehydrogenase level, 530 IU/l (normal range, 200–450 IU/l), blood urea nitrogen, 12.3 mg/dl (normal range, 8.0–20 mg/dl); creatinine, 1.12 mg/dl (normal range, 0.5–1.3 mg/dl); serum sodium, 138 mEq/l (normal range, 136–146 mEq/l); serum potassium, 3.0 mEq/l (normal range, 3.5–5.0 mEq/l); and chloride level, 97 mEq/l (normal range, 98–110 mEq/l). In addition, metabolic alkalosis was observed in the arterial blood gas analysis test (pH 7.483; partial pressure of CO_2_ [pCO_2_]_,_ 42.3 mmHg; pO_2_, 95.8 mmHg; HCO_3_, −31.0 mmol/l; and base excess, 7.6 mmol/l).

**Figure 1:**
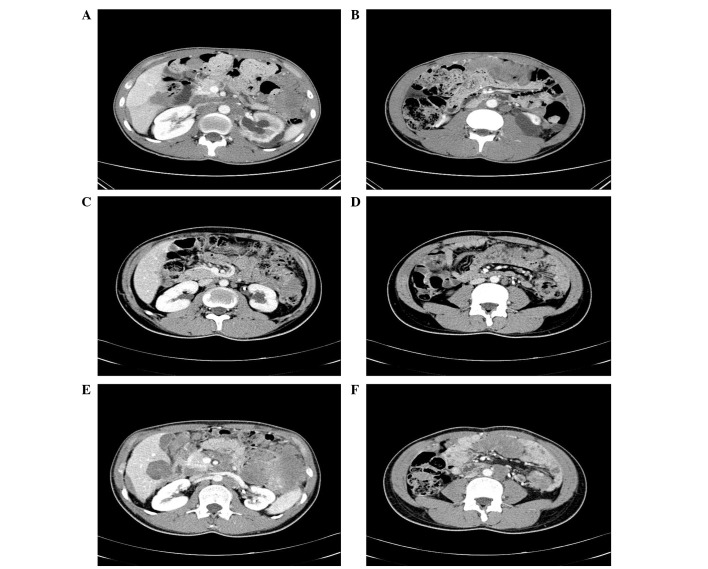
Abdomen and pelvis computed tomography prior to and following treatment. (A and B) Conglomerated lymph nodes (LNs) were observed along the aorta and inferior vena cava, as well as in the mesentery. Hydronephrosis of the left kidney and peritoneal seeding were also observed. (C and D) Conglomerated LNs and the peritoneal mass showed a decrease in size and the hydronephrosis of the left kidney had improved following the second cycle of chemotherapy. (E and F) Disease progression was observed following completion of chemotherapy.

Computed tomography (CT; Fig. 1) revealed multiple large masses that were composed of fused lymph nodes (LNs) of the mesenteric, paraaortic and inferior vena cava, as well as metastatic nodules in the liver, spleen and intraperitoneal space (Fig. 1A and B).

**Figure 2:**
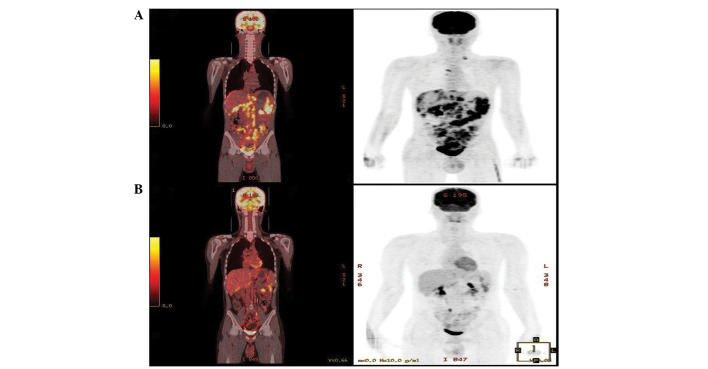
Positron emission tomography-computed tomography (A) prior to and (B) following treatment.

Hypermetabolism was observed by positron emission tomography-CT in the left supraclavicular LN, right internal mammary artery, retrosternal area and conglomerated LNs of the mesentery, aortocaval, paraaortic and pericaval areas (Fig. 2A). Physical and imaging examinations indicated the malignant nature of the tumor to be consistent with malignant lymphoma or a germ cell tumor.

**Figure 3:**
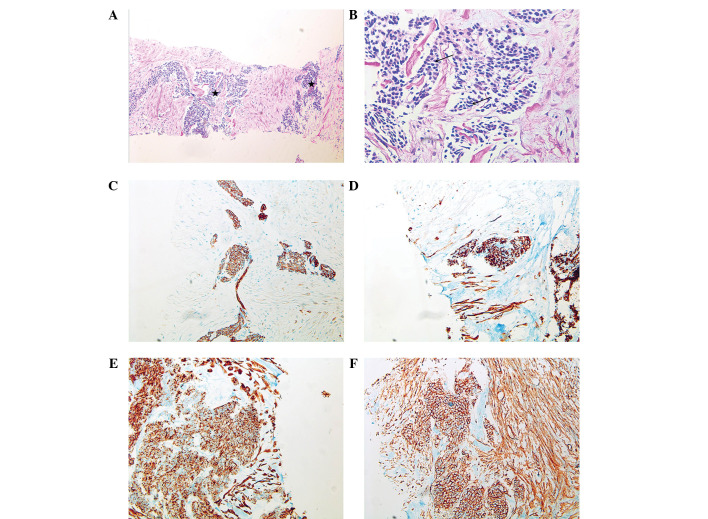
Desmoplastic small round cell tumor. (A) On low-power field (magnification, ×100), sheets and nests of small round, ovoid cells with minimal cytoplasm and hyperchromatic nuclei were noted (black star). (B) On high-power field (magnification, ×400), sheets and nests of small round, ovoid cells (arrows) with minimal cytoplasm and hyperchromatic nuclei were noted. (C–F) Imunohistochemical staining was positive for (C) cytokeratin, (D) desmin, (E) vimentin and (F) cluster of differentiation 99.(magnification, ×200).

First, a percutaneous needle biopsy of a palpable peritoneal nodule was performed. The biopsy specimen revealed the presence of a poorly differentiated tumor of variable size and shape composed of nests of small round cells surrounded by a prominent desmoplastic stroma (Fig. 3A and B). Immunohistochemically, the tumor cells coexpressed an epithelial marker (cytokeratin), a mesenchymal marker (desmin and vimentin), chromogranin A, and cluster of differentiation (CD) antigens, CD99 and CD56 (Fig. 3C–F). The sample was negative for Wilms’ tumor 1 (WT-1) protein and synaptophysin. Upon Ewing sarcoma (EWS)-fluorescence *in situ* hybridization analysis, the EWSR1 gene (22q12) rearrangement was detected in 93% of the cells.

Thus, malignant cells of the small round cell type, which were expressing CD99, desmin, cytokeratin and the EWSR1 gene (22q12) rearrangement were identified, as well as a malignant mass that was predominantly in the abdominal area of the young, male patient.

**Figure 4:**
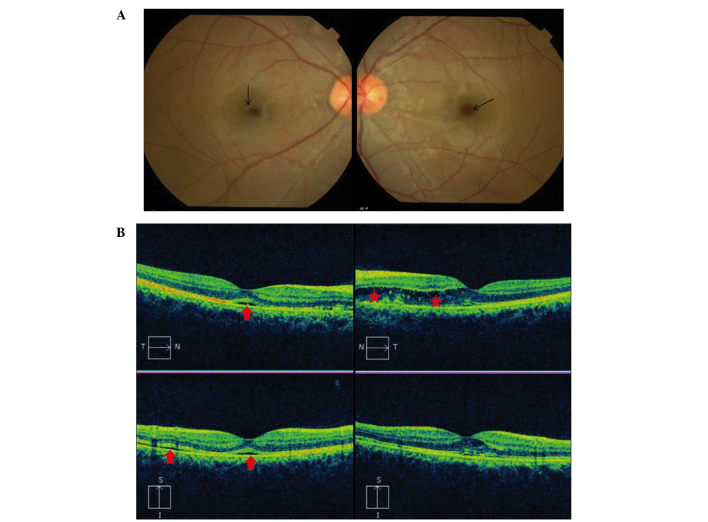
(A) Fundus photography revealed macular edema in each eye (arrow). A white lesion was observed in the macula (left) and a white spot was observed in the central fovea (right). (B) Optical coherence tomography demonstrated macular edema of the left eye (stars) and detachment of the sensory retina in the macular of the right eye (arrows) (left column, right eye; right column, left eye). T, temporal; N, nasal; I, inferior; S, superior.

**Figure 5:**
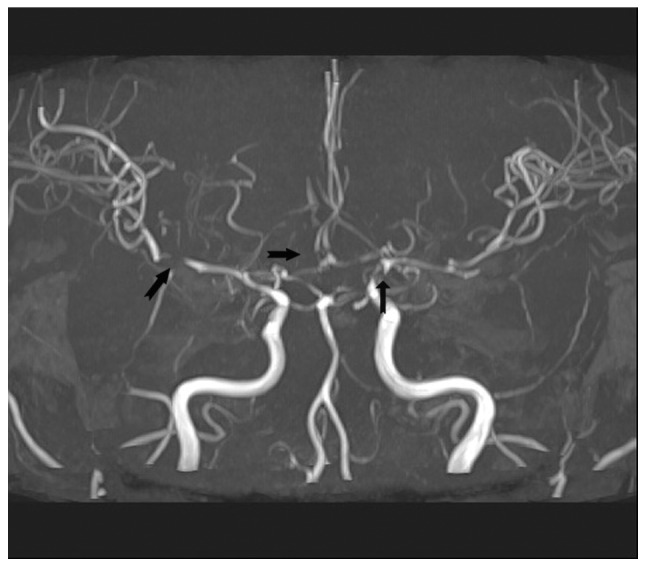
Brain magnetic resonance image demonstrates the blood vessel damage (arrows) due to the severe hypertension.

A diagnosis of DSRCT was determined based on these results, and the patient was treated with multiagent chemotherapy using the VAC/IE regimen (vincristine, adriamycin, cyclophosphamide, ifosfamide and etoposide). Vincristine 2 mg on day 1 for every three weeks, cyclophosphamide 900 mg/m^2^ on day 1 every three weeks, adriamycin 37.5 mg/m^2^ on day 1 and 75 mg/m^2^ on day 2 every three weeks. Subsewuently, etoposide 100 mg/m^2^ was administered on days 1–5 every three weeks and ifosfamide 1,800 mg/m^2^ was administered on days 1–5 every three weeks. However, the patient complained of a sudden visual disturbance during hospitalization, and therefore, underwent an ophthalmic examination and brain magnetic resonance imaging (MRI). During the ophthalmic examination, macular edema and macular detachment were observed, which were considered to be secondary hypertensive changes (Fig. 4A and B). The brain MRI did not demonstrate acute bleeding, or cerebral or cerebellum infarction; however, blood vessel damage due to severe hypertension was revealed (Fig. 5).

Additional hormone tests were performed during the secondary hypertension evaluation due to the severe hypertension (BP, 180/110 mmHg), hypokalemia, metabolic alkalosis, and hypertensive ophthalmic and cerebral vascular changes that were observed in this young patient that did not have a family history of hypertension.

Serum renin activity was 31.9 ng/ml/h in the supine position (normal range standing, 1.3–4.0 ng/ml/h and normal range in supine position, 0.15–2.33 ng/ml/h), while the aldosterone level was greatly increased to 64 ng/dl (normal range standing, 4.0–31.0 ng/dl and normal range in supine position, 1.0–1.6 ng/dl). The patient’s urinary vanillylmandelic acid level was 5.8 mg/day (normal range, 0–8 mg/day), metanephrine was 0.7 mg/day (normal range, 0–1.0 mg/day) and cortisol was 75.2 μg/day (normal range, 20–90 μg/day); these levels were all within the normal range. Doppler sonography of the kidney revealed normal blood flow without renal artery stenosis and a CT examination revealed no masses or vessel abnormalities in the kidney. However, mild hydronephrosis was observed in the left kidney. The 24-h BP monitoring revealed an average BP of 167/123 mmHg.

These findings were strongly indicative of a renin-secreting DSRCT. The patient was prescribed an aldosterone antagonist (spironolactone; 100 mg), an angiotensin II antagonist (olmesartan; 20 mg) and a calcium channel blocker (amlodipine; 5 mg). Upon treatment, the patient’s systolic BP decreased to 100–120 mmHg.

**Figure 6:**
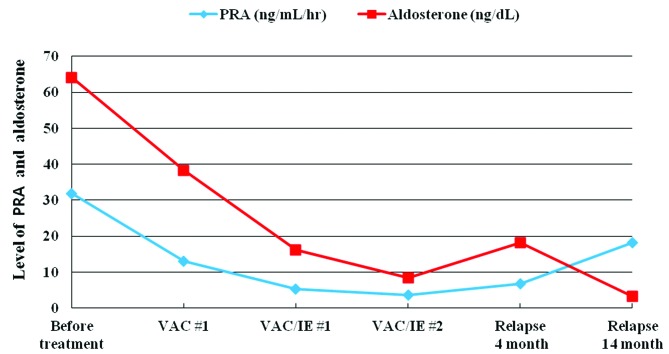
Changes in the renin and aldosterone levels following chemotherapy. PRA, plasma renin activity; VAC/IE, vincristine, adriamycin, cyclophosphamide, ifosfamide and etoposide.

Following a second cycle of the VAC/IE regimen, a partial response was achieved (Fig. 1C and D). The renin and aldosterone levels were gradually decreased to the normal range (renin activity decreased from 31.9 to 5.2 ng/ml/h, while the aldosterone levels decreased from 64 to 16.2 ng/dl). The patient’s BP was normalized without antihypertensive medication (Fig. 6).

The patient was treated with a total of six cycles of the VAC/IE regimen and achieved a partial response (Fig. 2B). However, further chemotherapy was not administered due to the patient’s asymptomatic state and concerns that the patient was too weak to receive further chemotherapy. At this time, the patient stopped attending the Chosun University Hospital.

**Figure 7:**
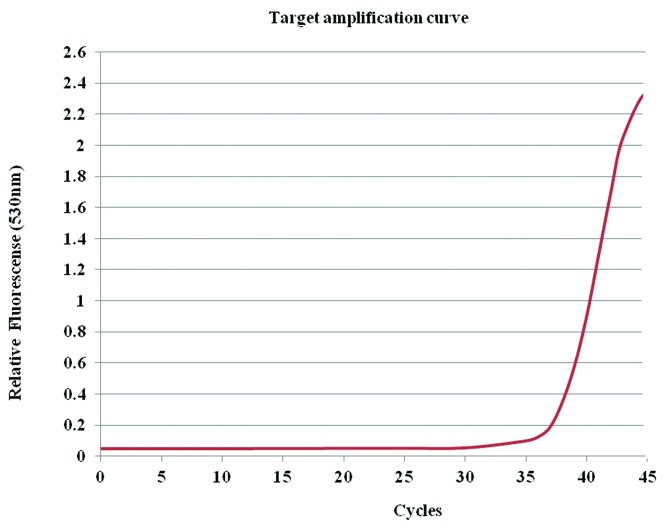
Reverse transcription quantitative polymerase chain reaction.

Following a four-month break from chemotherapy, the patient was readmitted to the Chosun University Hospital complaining of abdominal discomfort. It was identified that the tumor size had increased (Fig. 1E and F) and the renin level was re-elevated (Fig. 6). Based on these results, it was determined that the disease had progressed following cessation of chemotherapy. Second-line chemotherapy was recommended, however, the patient refused further chemotherapy. Subsequently, supportive therapy was administered, which included paracentesis. The focus of the present case was adjusted, and the aim was to provide direct evidence that the DSRCT was renin-secreting. Therefore, using malignant cells obtained from the patient’s ascites, reverse transcription quantitative polymerase chain reaction (RT-qPCR) analysis was performed. Total RNA was extracted using TRI reagent (RNAiso Plus; Takara Bio, Inc., Shiga, Japan) and cDNA was synthesized by RT (Invitrogen Life Technologies, Carlsbad, CA, USA) according to the manufacturer’s instructions. The cDNA was amplified by RT-qPCR using a Roche LightCycler 2.0 system (Roche Diagnostics, Mannheim, Germany) for 45 cycles. The PCR reaction contained 4 μl cDNA (diluted to 1:5), 4 ml MgCl_2_, 10 pmol of each primer and 4 μl Fast Starter Mix buffer (4 mM deoxynucleotide triphosphates, 2X SYBR^®^ Green dye and 2.5 U Taq polymerase). The primers and conditions used for RT-qPCR are shown in Tables I and II. RT-qPCR revealed that the patient’s ascites contained renin (Fig. 7). At 16 months, following relapse, the patient refused further chemotherapy and succumbed.

## Discussion

DSRCT is a rare and aggressive malignant neoplasm that occurs in adolescents and young adults. The mean age at diagnosis is ~22 years, and ranges between six and 49 years; the male to female ratio is 4:1 (5). Clinical manifestations are often associated with widespread abdominal disease and distant metastases are frequently present at the time of diagnosis. Symptoms include abdominal pain and distension and potentially nausea or emesis. DSRCT is a member of the large family of small round cell tumors of childhood, along with the primitive neuroectodermal tumor, alveolar and embryonal rhabdomyosarcoma, and poorly differentiated synovial sarcoma and rhabdoid tumors. However, DSRCT is characterized by a distinct immunohistochemical pattern and a recurrent, specific chromosomal translocation (5–7). The tumor is composed of desmoplastic stroma with nests of small round blue cells, which show immunohistochemical reactivity for epithelial (cytokeratin and epithelial membrane antigen), mesenchymal (vimentin), neural (neuron-specific enolase) and muscle (desmin) markers when visualized using light microscopy (5). DSRCT is associated with a unique chromosomal translocation, t(11;22)(p13;q12), that fuses the N-terminus of the EWS gene to the C-terminus of the WT-1 gene (6,7). The presence of this translocation provides confirmation of the diagnosis of DSRCT. The current case presented the characteristic morphological, immunohistochemical (cytokeratin, vimentin, desmin and CD99) and molecular features of DSRCT.

Despite their rarity, paraneoplastic syndromes must always be considered in cancer patients with unusual clinical findings. The current patient exhibited hypokalemia at presentation, therefore, an endocrine cause for the hypertension was sought out. Serum renin and aldosterone were found to be markedly elevated, which explained the abnormal clinical findings. The juxtaglomerular apparatus predominantly secretes renin into the interstitium of the kidney, and not the lumen of the vessel. In addition to the kidney, renin has also been identified in a number of organs, including the brain, genital tract, salivary gland, vessels, skeletal muscle and heart. Therefore, tumors arising from these organs are capable of secreting renin. Renin-secreting tumors are classified into the following three groups: i) Tumors arising from the juxtaglomerular apparatus of the kidney; ii) renin-secreting renal tumors, including Wilms’ tumor, clear cell-type renal cell carcinoma, oncocytoma and mesoblastic nephroma; and iii) extrarenal tumors, including granulosa cell tumors, lung cancer and pancreatic cancer (8–11). In total, ~40 patients with a renin-producing tumor (excluding lesions of the juxtaglomerular apparatus) had been reported by 1988 and the majority were of renal origin. The renin-secreting tumor triad consists of hypertension, hypokalemia and elevated plasma renin activity (PRA). Hypertension in patients with an extrarenal tumor is more severe than hypertension in patients with a renal tumor. In the present patient, the BP was 180/110 mmHg, the serum potassium level was 3.0 mEq/l (normal range, 3.5–5.0 mEq/l) and the PRA had increased by 8–10 times the upper limit of the normal range (normal range standing, 1.3–4.0 ng/ml/h and normal range in supine position, 0.15–2.33 ng/ml/h). In addition, the patient complained of visual disturbance resulting from the hypertensive retinopathy. The elevated renin level at presentation fell following successful treatment of the primary tumor, which strongly indicates that the tumor was the source of the renin. The diagnosis of a renin-secreting tumor is usually determined by positive immunostaining of renin or by electron microscopic identification of renin granules (13–16). The predominant characteristic finding from electron microscopy are two types of granules: Rhomboid crystalline protogranules and amorphous homogeneous, round, electron dense mature granules (13).

However, immunostaining and electron microscopic identification are not readily available in clinical practice and the use of these methods in the context of this case report is experimental and not universal.

In conclusion, although immunostaining for renin or electron microscopic examination were not performed in the present study, the diagnosis of a renin-producing tumor was determined due to the elevated serum renin and aldosterone levels, which normalized during tumor regression and were re-elevated during tumor progression. In addition, the biosynthesis of renin was confirmed by the demonstration of mRNA in the patient’s ascites that codes for the renin precursor. To the best of our knowledge, the current report is the first to demonstrate an extremely rare case of renin secretion from a rare type of cancer termed DSRCT. The results of the current study indicated that DSRCT may be considered as one of the differential diagnoses for extrarenal renin producing tumors.

## Figures and Tables

**Table I:** Primers for reverse transcription quantitative polymerase chain reaction.

Gene	Primer
Renin	Sense: tga cac tgg ttc gtc caa tg Antisense: agc tgg agg aat ccg aag c
β-actin	Sense: gac tat gac tta gtt gcg tt Antisense: gtt gaa ctc tac ata ctt ccg

**Table II:** Conditions for reverse transcription quantitative polymerase chain reaction.

Gene	Hot start	Denaturation	Annealing	Extension
Renin	95°C, 10 min	95°C, 15 sec	60°C, 5 sec	72°C, 10 sec
β-actin	95°C, 10 min	95°C, 15 sec	55°C, 5 sec	72°C, 21 sec
